# Assessment of Dentists' Knowledge of Space Maintainer Indications After Primary First Molar Extraction: A Cross-Sectional Study

**DOI:** 10.7759/cureus.61738

**Published:** 2024-06-05

**Authors:** Farah Babakurd, Nawaf H Al Shammary, Lilian Azrak, Zuhair Al-Nerabieah, Muaaz Alkhouli, Mayssoon Dashash

**Affiliations:** 1 Pediatric Dentistry, Damascus University, Damascus, SYR; 2 Orthodontics, University of Ha'il, Ha'il, SAU

**Keywords:** children, pediatric dentistry, dental education, curriculum, space maintainers

## Abstract

Background

Space maintainer appliances can potentially reduce the need for extensive orthodontic treatment in the future for children. However, they can also lead to various complications, such as an increased risk of dental caries and gingival diseases. It is crucial for dentists to carefully evaluate a decision each child's specific situation before deciding whether to apply a space maintainer or not. This study aimed to evaluate the knowledge of dentists at Damascus University, both at undergraduate and specialist levels, regarding when space maintainers should be recommended.

Methods

A cross-sectional study was conducted involving 158 dentists (151 at the undergraduate level and seven at the specialist level). The questionnaire included 13 questions, covering demographic information and self-assessment, and 10 scenarios that are commonly encountered in clinical practice.

Results

The response rate was 50%. A majority of the participants (85, 53.8%) demonstrated inadequate knowledge. No significant correlations were found between knowledge level and gender (P = 0.853), practice experience (P = 0.171), or self-assessment (P = 0.383).

Conclusions

Most dentists exhibited a lack of knowledge about space maintainers, with no correlation identified between knowledge level, gender, practice experience, and self-assessment. It is recommended that educational curricula be updated to ensure that clinicians have a better understanding of when to use space maintainers in children.

## Introduction

In pediatric dentistry, preserving the primary teeth is paramount, as they serve critical functions in a child's oral health and development. The adage "the child's primary tooth is the best space maintainer" underscores the significance of the primary teeth in guiding the eruption of permanent teeth and maintaining proper alignment [1].

Despite advances in preventive measures, such as fluoride treatments and sealants [2], recent epidemiological studies conducted in Syria have unveiled a troubling surge in the Decayed, Missing, and Filled Teeth (DMFT) indices [3,4]. Furthermore, it is noteworthy to mention that a recent study revealed a high prevalence of molar incisor hypomineralization (MIH) [5].

This alarming trend underscores the need for a deeper understanding of the factors contributing to early primary tooth loss and the subsequent implications for long-term oral health outcomes [6]. Space maintainers play a crucial role in preserving the integrity of the dental arch following the premature loss of the primary teeth. By preventing adjacent teeth from drifting into the vacant space and potentially impeding the eruption of permanent successors, space maintainers help preserve proper occlusion and reduce the likelihood of more extensive orthodontic treatment later in life [7,8].

However, the decision to place a space maintainer necessitates a thorough understanding of the indications and contraindications [9] and proficiency in the selection and placement of the appropriate device based on individual patient needs [10-12].

Given the pivotal role of dentists in managing pediatric dental care [13], it becomes imperative to gauge their knowledge and awareness regarding the indications for space maintenance following the extraction of primary first molars [14-16]. Damascus University stands as a focal point for dental education and training in Syria, making it an ideal setting to investigate the preparedness of both undergraduate students and specialist practitioners in addressing this critical aspect of pediatric dental care.

By assessing the decision-making process and treatment protocols employed by dentists when faced with cases requiring space maintenance [17,18], we can gain valuable insights into the alignment between theoretical knowledge and clinical practice. Such insights are essential for refining educational curricula, enhancing clinical training programs, and ultimately improving the quality of pediatric dental care delivery.

Through a comprehensive assessment of the knowledge base among dentists, this study aims to identify potential gaps in understanding and practice, with the ultimate goal of informing targeted interventions and educational initiatives. In addition to evaluating the theoretical understanding of space maintenance indications, this study will also explore the practical application of knowledge within clinical settings.

## Materials and methods

Ethical approval

Ethical approval was obtained from the Scientific Research Committee at Damascus University in Damascus, Syria (approval no. 2864, dated 26/11/2023). Informed consent was obtained from participants after providing them with detailed information about the study's objectives and procedures.

Sample size calculation

A total of 424 dentists were surveyed at Damascus University, comprising 400 undergraduate students and 24 specialists. The sample size was determined using the CheckMarket survey tool (Medallia, USA) [19]. To ensure representation and statistical validity the study aimed for a minimum sample of 158 participants, including 151 undergraduates and seven specialists, to achieve a 95% confidence level with a 5% margin of error. To accommodate an estimated 50% response rate, 316 invitations were necessary, distributed among 302 undergraduates and 14 specialists.

Study design

A cross-sectional study was undertaken to assess dentists' understanding at Damascus University, spanning both undergraduates and specialists, particularly focusing on space maintainer indications.

A 13-question questionnaire was formulated in Arabic after a thorough literature review. The questionnaire consisted of two parts: the first section captured demographic information and self-assessment, while the second part consisted of 10 scenarios representing common clinical cases dentists encounter.

Expert validation confirmed the questionnaire's content, with question importance assessed using the Aiken's Index [20]. Aiken's Index, also known as Aiken's V, is a measure of content validity that evaluates how well items represent the construct they aim to measure. This index is calculated based on ratings provided by a panel of experts, with values ranging from 0 to 1. Higher values indicate greater content validity. For this study, each item in the questionnaire was rated by a group of experts on a scale from 1 to 5, and items with an Aiken's V value of 0.80 or higher were considered to have high content validity.

A pilot test involved 10 undergraduate and 10 specialist volunteers, confirming the questions' clarity, relevance, and overall effectiveness. Questionnaire reliability was assessed using Cronbach's alpha test [21], yielding a range of 0.75 and 0.87, with a median of 0.81, denoting strong reliability.

Participants were recruited from Damascus University, specifically targeting both undergraduate dental students and specialists in pediatric dentistry. The recruitment process involved sending invitations via email to all eligible participants within the university. The email included a brief overview of the study's objectives, the importance of the research, and instructions for completing the questionnaire.

The questionnaire was introduced to the participants through an online survey platform. The email invitation contained a link to the survey, which began with an informed consent form. The participants were required to read and agree to the consent form before proceeding to the questionnaire. The questionnaire was designed to be concise and user-friendly, ensuring that participants could complete it within a reasonable time frame. In addition, reminders were sent out two weeks after the initial invitation to encourage participation and improve the response rate.

These measures ensured that participants were well-informed about the study and motivated to contribute, thereby enhancing the quality and reliability of the data collected.

Data collection spanned December 2023 to January 2024, offering participants the choice of voluntary and anonymous participation Knowledge questions were scored either correct or incorrect according to the Advanced Education in Pediatric Dentistry (AEPD) and American Academy of Pediatric Dentistry (AAPD) recommendations, with one point for correct and 0 for incorrect answers [22]. Overall knowledge scores were classified into three tiers as follows: 1) poor score: <50% correct, 2) fair score: 50-75% correct, and 3) satisfactory score: >75% correct.

Collected Google Forms data were imported into an Excel spreadsheet, with statistical analysis undertaken using IBM SPSS Statistics for Windows, version 23.0 (released 2015, IBM Corp., Armonk, NY). Descriptive statistics, including frequency and percentage distribution, were calculated. The association between categorical variables, such as knowledge level, gender, practice, and self- assessment, was determined using chi-square, with statistical significance set at 0.05 (P < 0.05).

## Results

Out of the 316 dentists invited to participate in the study (302 undergraduates and 14 specialists), 158 responded, yielding a response rate of 50%. Among the respondents, 94 (59.5%) were female, and the majority (151, 95.6%), were undergraduate students. A notable 130 (82.3%) participants self-assessed their knowledge (Table 1).

**Table 1:** Demographic characteristics of the participants.

Characteristics	N (%)
Gender	
Female	94 (59.5)
Male	64 (40.5)
Practice	
Undergraduate	151 (95.6)
Specialized	7 (4.4)
Self-assessment	
Proficient	6 (3.8)
Average	130 (82.3)
Poor	22 (13.9)

The majority of the participants (148, 93.7%) believed in the necessity of applying space maintainers for the early loss of the first primary molar. However, 84 (53.2%) of the respondents overlooked multiple factors affecting dental arches during the transition from primary to permanent occlusion. Only 29 (18.4%) suggested not using space maintainers when the child's oral health was poor.

A total of 97 (61.4%) participants correctly identified the need for space maintainers when the molar relationship develops into a Class III malocclusion. In addition, 103 (65.2%) recognized the importance of applying a space maintainer when the molar relationship evolves into a distal step terminal plane, indicative of a Class II malocclusion. Furthermore, a significant majority (111, 70.3%) acknowledged the necessity of space maintainer application in instances of crowding in the mandibular anterior teeth, even following the eruption of the permanent lower first molar.

More than half of the participants (121, 76.6%) were unsure about the duration of lingual arch application after the premature loss of the mandibular primary canine. A majority (106, 67.1%) indicated that space maintainers should be applied at age 3 before the effective eruption of permanent molars. In addition, 93 (58.9%) preferred using a band and loop to prevent palatal movement of the incisors, and 117 (74.1%) suggested using a space maintainer in the maxilla despite the eruption of the permanent first molar (Table 2).

**Table 2:** Knowledge regarding the indication for space maintainers. Correct answers are written in bold.

Questions	N (%)
A five-year-old child had premature extraction of the upper left temporary molar. After a follow-up of 81 months, the case showed no crowded permanent successors or canine block-out at the extraction site. This can be explained by:	
Preventive orthodontics was performed using space maintainers.	148 (93.7)
The increase in the dimensions of the dental arch is sufficient to compensate for the expected distance loss.	10 (6.3)
A five-year-old child had premature extraction of the upper left temporary first molar. After a follow-up of 81 months, the case showed crowded permanent successors at the control site compared with the extraction site. This can be explained by:	
not applying a space maintainer on the left side when extracting the upper temporary molar.	84 (53.2)
		Other factors related to facial growth, oral habits, etc.	74 (46.8)
Extracting the temporary lower first molar was performed (non-restorable due to carious injury). The following procedure is indicated:			
Apply a space maintainer.	129 (81.6)
Space maintainers were not indicated due to poor oral hygiene.	29 (18.4)
		In a seven-year-old child, the molar relationship in the terminal plane mesial step has developed to a third dental class. If the upper temporary first molar is extracted on one side, then it is indicated:	
Apply a space maintainer.	61 (38.6)		
There is no need to apply for a space maintainer.	97 (61.4)
In a 7-year-old child, the molar relationship in the terminal plane distal step has developed to a second dental class, if the temporary upper first molar was extracted on one side, then it is indicated:	
		Apply a space maintainer.	103 (65.2)
There is no need to apply for a space maintainer.	55 (34.8)
In a 7-years old child with good oral hygiene, has a crowding in the anterior teeth with extracted temporary first molar after the eruption of the permanent lower first molar, the adequate indication is:	
		Apply a space maintainer.	111 (70.3)
There is no need to apply for a space maintainer.	47 (29.7)
The early loss of the temporary lower canine is an indicator of possible crowding; therefore, to maintain asymmetry, it's recommended to extract the opposite temporary canine, followed by:	
		Applying a lingual arch to prevent losing the length of the dental arch until the eruption of the first permanent second molars.	37 (23.4)
Applying a lingual arch to prevent losing the length of the dental arch until the eruption of the first permanent premolars.	121 (76.6)		
		A three-year-old child (before the effective eruption of the permanent first molar) has lost the upper temporary first molar. The recommended procedure for this age:	
Apply a space maintainer.	106 (67.1)
There is no need to apply for a space maintainer.	52 (32.9)
One of the possible complications to the early extraction of the temporary upper first molars is the palatal inclination of the incisors; therefore, it is indicated to:	
		Applying palatal arch.	65 (41.1)
Applying crown and loop for each side.	93 (58.9)		
		A seven-year-old child lost his temporary upper first molar after the eruption of the permanent first molar. Is there any indication for space maintainers:	
No, until the eruption of the permanent first molar.	41 (25.9)
		Yes, to avoid a 1 mm loss in the upper arch.	117 (74.1)

Most participants (85, 53.8%) demonstrated a weak level of knowledge regarding space maintainer indications (Table 3). There was no significant correlation between knowledge level and gender (P = 0.853), practice experience (P = 0.171), or self-assessment (P = 0.383) (Table 4).

**Table 3:** Level of knowledge regarding the indication for space maintainers.

Level of knowledge	N (%)
Poor level of knowledge	85 (53.8)
Fair level of knowledge	73 (46.2)
Satisfactory level of knowledge	0 (0)

**Table 4:** Association between the level of knowledge, gender, practice experience, and self-assessment.

		Level of knowledge			p-value	
		Poor level of knowledge N (%)	Fair level of knowledge N (%)			
Gender	Female	50 (31.6)	44 (27.8)	0.853		
	Male	35 (22.2)	29 (18.4)			
Practice	Undergraduate	83 (52.5)	68 (43.0)	0.171		
	Specialized	2 (1.3)	5 (3.2)			
Self-assessment	Proficient	2 (1.3)	4 (2.5)	0.383		
	Average	73 (46.2)	57 (36.1)			
	Poor	10 (6.3)	12(7.6)			

## Discussion

The investigation into space maintainers within pediatric dentistry aims to understand clinicians' knowledge regarding the indications for these essential devices following the extraction of primary first molars. Conducted at the Department of Pediatric Dentistry, Damascus University, this study stands as a significant research endeavor in the region, shedding light on critical aspects of pediatric dental care.

The scenarios in the questionnaire provided valuable insights into the participants' understanding of space maintainer indications. The scenario with the highest correct response rate involved a seven-year-old child with good oral hygiene, experiencing crowding in the anterior teeth and an extracted temporary first molar after the eruption of the permanent lower first molar. In this case, 111 participants (70.3%) correctly indicated that applying a space maintainer is necessary. This high correct response rate suggests that participants are well-informed about the importance of maintaining space in cases where crowding and subsequent tooth eruption complications are likely. The awareness that space maintainers help in preventing further malocclusion and support the proper alignment of permanent teeth highlights the effectiveness of current educational approaches in certain aspects of pediatric dental care.

Conversely, the scenario with the most incorrect answers involved a five-year-old child who had a premature extraction of the upper left temporary molar, followed by an 81-month follow-up that showed no crowded permanent successors or canine block-out at the extraction site. A significant majority of participants (148, 93.7%) incorrectly attributed the favorable outcome to preventive orthodontics using space maintainers. Only 10 participants (6.3%) correctly recognized that the increase in the dimensions of the dental arch could be sufficient to compensate for the expected distance loss. This misconception indicates a gap in understanding the natural compensatory mechanisms of dental arch growth and the circumstances where space maintainers may not be necessary. It underscores the need for improved education on the natural development of the dental arch and the specific indications for space maintainer use to ensure that practitioners can make well-informed decisions in varied clinical scenarios.

The findings reveal a prevalent trend of insufficient knowledge among participants regarding space maintainer indications. Both undergraduate dental students and pediatric dentists displayed a concerning propensity toward a weak level of understanding. These findings resonate with similar investigations conducted elsewhere, which also highlighted inadequate knowledge among general and specialist dentists concerning the immediate management of tooth extractions [23].

In addition to assessing clinicians' knowledge of the immediate management of tooth extractions, it is essential to consider the different types of space maintainers and the factors influencing their planning. Space maintainers play a crucial role in pediatric dentistry, ensuring proper space for the eruption of permanent teeth following the premature loss of primary teeth. The main types of space maintainers include fixed maintainers like band and loop, crown and loop, and distal shoe; removable maintainers such as acrylic removable appliances and Hawley appliances; and functional maintainers like the lingual arch and Nance appliance. Each type has specific indications based on the location and number of missing teeth, the age of the child, and their ability to maintain oral hygiene.

Several factors affect the planning and selection of space maintainers. These include the child's age, the type and number of missing teeth, oral hygiene capabilities, cooperation level, current occlusal relationship, and dental development stage. In addition, a thorough analysis of arch length and available space is critical to ensure the space maintainer effectively preserves the necessary space for future permanent teeth. Understanding these factors is essential for dental professionals to provide optimal care, emphasizing the need for comprehensive education and training in the application and management of space maintainers in pediatric dentistry.

Moreover, the absence of statistically significant differences in knowledge levels based on participant characteristics, including gender, practice experience, and self-assessment, underscores the systemic nature of the issue [24-26].

The study's alignment with findings from a recent study, which demonstrated no correlation between gender and academic performance, further reinforces the notion that deficiencies in space maintainer knowledge transcend individual demographics [27].

The implications of these findings extend into the realm of clinical practice and patient care. As was demonstrated in an earlier study, pediatric dentists exhibited a higher level of knowledge than newly graduated dentists regarding the management of extracted teeth, underscoring the pivotal role of ongoing education and training in shaping clinical competency [28]. Similarly, the study's emphasis on the urgent need for integrating space maintainer science into the curriculum of the Pediatric Dentistry Department at Damascus University echoes the sentiments expressed by Yan et al., who highlighted the importance of updating educational curricula to address knowledge gaps among dental students [29].

Despite the valuable insights gleaned from this study, it is imperative to acknowledge its limitations. The reliance on clinical images, rather than radiographs, for assessing space maintainer planning may have constrained the comprehensive evaluation of this multi-factorial process. Future research endeavors would benefit from incorporating a more holistic approach, encompassing diverse diagnostic modalities to enhance the accuracy and validity of findings. Another limitation of this study includes the type of space maintainer suggested based on the clinical scenario questions. The questionnaire did not differentiate adequately between scenarios requiring fixed versus removable space maintainers, which could impact the participants' responses and the interpretation of their knowledge. Fixed space maintainers, such as band and loop or distal shoes, and removable maintainers, like acrylic appliances, have different indications and practical applications. Without clear distinctions in the questionnaire, the responses may not accurately reflect the clinicians' understanding and decision-making process for each specific type of maintainer in various clinical situations. This limitation highlights the need for more detailed and scenario-specific questions in future studies to better assess the knowledge and decision-making skills of dental professionals regarding space maintainer use.

## Conclusions

This study highlighted significant gaps in knowledge among dental professionals regarding the indications for space maintainers following the extraction of primary first molars. It was evident that many participants demonstrated inadequate knowledge. The analysis showed no significant correlation between knowledge level and gender, practice experience, or self-assessment.

A majority of participants believed in the necessity of space maintainers for early loss of the first primary molar, yet many overlooked multiple factors affecting dental arches during the transition from primary to permanent occlusion. Furthermore, a substantial portion recognized the importance of space maintainers in cases indicative of Class II malocclusion, and many acknowledged their necessity in instances of crowding in the mandibular anterior teeth, even after the eruption of the permanent lower first molar.

This research is important as it underscores the urgent need for enhanced education and training in the application of space maintainers within the dental curriculum. By addressing these knowledge gaps, dental professionals can improve their clinical practices, thereby ensuring better oral health outcomes for pediatric patients. Enhanced understanding and appropriate use of space maintainers are critical for preventing malocclusion, preserving dental arch length, and supporting the proper eruption of permanent teeth.
